# Psychometric properties of the purpose in life scale in Peruvian university students

**DOI:** 10.3389/fpsyg.2026.1771160

**Published:** 2026-03-06

**Authors:** Vilma Vilca-Pareja, Rodrigo-Alejandro Ardiles-Irarrázabal, Manuel Edmundo Hillpa-Zuñiga, Victor Ritchar Yana-Calla

**Affiliations:** 1 Escuela de Posgrado, Estudios Generales, Universidad Católica de Santa María, Arequipa, Peru; 2 Department of Nursing, University of Antofagasta, Antofagasta, Chile; 3 Faculty of Administration and Business, Universidad Tecnológica del Perú, Lima, Peru; 4 Escuela Profesional de Psicología, Universidad Católica de Santa María, Arequipa, Peru

**Keywords:** emerging adults, eudaimonic wellbeing, PILEA-4, positive youth development, psychometric validation, purpose in life, university students

## Abstract

**Introduction:**

Purpose in Life is a key psychosocial resource during the university stage, contributing to psychological adjustment and well-being. To date, no psychometric validations of the abbreviated Purpose in Life scale (PILEA-4) have been reported in Latin America. This study aimed to examine the psychometric properties of the PILEA-4 in a sample of Peruvian university students.

**Methods:**

A total of 1,006 students (55.5% women; ages 17–30) from public and private universities in Arequipa, Peru, participated. The PILEA-4 (four Likert-type items) and a Positive Youth Development questionnaire (5Cs) were administered. Due to multivariate non-normality, a unidimensional model was estimated through confirmatory factor analysis using robust maximum likelihood. Model fit was evaluated using χ^2^, df, CFI, TLI, SRMR, and RMSEA with confidence intervals. Reliability (omega) and convergent validity (average variance extracted and correlations with the 5Cs) were examined. Measurement invariance across sex was tested through multigroup analysis.

**Results:**

The unidimensional model showed good global fit (CFI = 0.985; TLI = 0.956; SRMR = 0.023; RMSEA = 0.079, 95% CI 0.054–0.108), with standardized loadings ranging from 0.69 to 0.90. The scale demonstrated high reliability (ω = 0.873) and adequate convergent validity (AVE = 0.641; correlations with the 5Cs ranged from 0.19 to 0.57). Multigroup analyses confirmed measurement invariance across sex up to the strict level, supporting valid comparisons between women and men

**Discussion:**

These findings provide solid psychometric evidence supporting the use of the PILEA-4 as a brief measure for screening and monitoring purpose in life in Peruvian university settings. To our knowledge, this represents the first validation of the PILEA-4 reported in Latin America.

## Introduction

University life presents multiple challenges. University students face personal, family, and academic demands such as romantic breakups, separation from their family of origin, and adaptation to a new educational environment. These challenges may result in stress, anxiety, and depression. Recent evidence indicates that the prevalence of suicide risk among university students reached 43.8% (Gómez-Tabares et al., 2024). Batanova et al. (n.d.) state that several factors influence mental health problems in young people: (a) lack of meaning, purpose, and direction (not knowing what to do with one’s life), (b) financial concerns and pressure to succeed, and (c) relational deficits, including the perception of not mattering to others and feelings of loneliness A well-established protective factor in this life stage is Purpose in Life (PIL). Humanistic psychology recognized the importance of purpose for coping with adversity (Frankl, 2011), and Positive Psychology has continued to expand this construct (Ehsani et al., 2024).

Purpose in Life has been defined as a central and self-organized life aim that guides goals, regulates behavior, and provides meaning to experience (Pérez-Díaz et al., 2024). Purpose in Life is also conceived as a vital objective that guides goals, regulates behavior, and gives meaning to life (McKnight and Kashdan, 2009).

Purpose in Life is considered a key indicator of eudaimonic wellbeing (Damon et al., 2003). Inverse relationships have been reported between purpose and indicators of psychological dysfunction such as depression and anxiety (Ryff and Keyes, 1995; Ryff, 2014; Boreham and Schutte, 2023). From a positive health perspective, purpose has been associated with perceived health (Campbell et al., 2024), and healthy lifestyle habits (Hill et al., 2019). Meaning in life and reasons for living act as protective factors against suicidal ideation and behavior (Kleiman and Beaver, 2013; Bakhiyi et al., 2016). Purpose in Life promotes life planning, motivation, persistence when facing obstacles, and strengthens resilience (Gómez-Tabares et al., 2024). In university settings, purpose is related to learning and service engagement that contributes to personal development (Lund et al., 2024).

In contrast, low purpose among university students is negatively associated with mental health (Lund et al., 2024), and with risk or antisocial behaviors. Although purpose is often considered a developmental milestone typically achieved in adulthood, research has demonstrated the importance of assessing it during emerging adulthood (Hill et al., 2016). It has also been shown that purpose in life is positively associated with self-esteem in adolescents (Sharma et al., 2023). According to Hill et al. (2016), purpose in life and identity develop simultaneously and can reinforce each other during emerging adulthood.

With respect to convergent validity, Purpose in Life has been associated with Positive Youth Development (PYD), particularly through the 5Cs model (*competence, confidence, connection, character, and caring*). Evidence from Peruvian university students indicates that PYD dimensions significantly predicted Purpose in Life (Rojas Zegarra et al., 2025). Likewise, research in Chilean youth reported positive and significant correlations between the 5Cs and Purpose in Life (Pérez-Díaz et al., 2024), and similar findings have been described in Malaysia (Abdul Kadir and Mohd, 2021).

Several measurement instruments have been created to evaluate Purpose in Life. Hill et al. (2016) validated the Purpose in Life Scale for Emerging Adults in American and Canadian samples, reporting adequate reliability, predictive validity, and construct validity in emerging adults. Psychometric properties of the Purpose in Life Scale (PIL) have also been evaluated in Spanish populations, indicating satisfactory validity and reliability (Rubio-Belmonte et al., 2022).

The abbreviated version of the Purpose in Life Scale for Emerging Adults (PiLEA) is an unidimensional instrument composed of four Likert-type items rated from “strongly disagree” to “strongly agree” (e.g., “My life has direction”) (Hill et al., 2016). In the sample used in that study, the questionnaire showed good reliability (*α* = 0.81). Despite these advances, there are still gaps in validation within the Peruvian context and in specific university populations (Caycho-Rodríguez et al., 2022). Although its use has been growing, we did not identify validations of the PILEA-4 in Latin America according to an exploratory search (Scopus, WoS, SciELO, Dialnet, Google Scholar; November 26, 2025). This gap motivates the present validation in Peruvian university students. Consequently, it is necessary to validate this instrument in the Peruvian context, since the availability of brief and valid measures is key to evaluating and implementing educational interventions aimed at promoting Purpose in Life during the university period (Zhu et al., 2024), given its protective function against mental health problems and risk behaviors such as suicide and substance use.

Although previous studies have examined purpose in life using longer or conceptually related instruments, to our knowledge, this is the first psychometric validation of the abbreviated PILEA-4 specifically in Latin American university students. Addressing this gap is particularly relevant given the growing interest in brief and scalable measures of eudaimonic wellbeing in higher education contexts. Therefore, the aim of the present study was to examine the psychometric properties of the PILEA-4 in a sample of Peruvian university students, including its factorial structure, reliability, and measurement invariance by sex.

## Method

### Participants

The study was conducted using a quantitative, cross-sectional, instrumental design. Non-probabilistic convenience sampling was used, with a total sample of 1,006 university students from different Peruvian universities. The sample included students of different sexes, allowing measurement invariance analyses. Participation was voluntary, and the inclusion criteria were being a regular higher education student and agreeing to participate by means of informed consent.

In order to justify the adequacy of the sample size for the confirmatory factor analysis, an *a priori* estimate of the minimum required size was made using Daniel Soper’s sample size calculator for structural equation models. This estimate considered the number of observed variables, the model structure, and an adequate level of statistical power. The results indicated that the minimum sample size required was substantially lower than the number of participants included in the study, so the final sample used far exceeds the minimum necessary for a stable and reliable estimation of the model parameters (Soper, n.d.).

### Instruments

#### Purpose in life (PILEA-4)

The PILEA-4 is a unidimensional scale consisting of 4 items rated on a Likert scale (1 = strongly disagree to 5 = strongly agree). An example item is: “I know which direction my life will follow” (Hill et al., 2016). To ensure linguistic appropriateness in Spanish, the Spanish adaptation and available psychometric evidence of Purpose in Life scales reported by Rubio-Belmonte et al. (2022) were considered as a reference, using their terminology and writing style as guidance for the formulation and adjustment of items in Spanish. This reference pertains only to linguistic considerations; the specific psychometric validation of the PILEA-4 is carried out in the present study.

To document regional background, an exploratory (non-systematic) search was conducted to identify psychometric validations of the PILEA-4 in Latin America (Scopus, Web of Science, SciELO, Dialnet, and Google Scholar; search terms: “PILEA-4” OR “Purpose in Life short” AND (validate* OR validation) AND (Latin* OR Spanish OR Portuguese); last search: November 26, 2025). No validation studies published in the region were identified.

#### Positive youth development – short form (PYD-SF)

This is a 34-item questionnaire that evaluates the 5Cs of Lerner’s model: Competence, Confidence, Character, Caring, and Connection (Geldhof et al., 2014). Items are rated on a 5-point Likert scale (1 = strongly disagree to 5 = strongly agree). The Spanish version with evidence of internal consistency and factorial structure in Spanish-speaking populations was used (Marín-Gutiérrez et al., 2024). In Peru, satisfactory alpha coefficients have been reported for all dimensions: competence (*α* = 0.73), confidence (α = 0.77), connection (α = 0.77), care (α = 0.88), and character (α = 0.59) (Manrique-Millones et al., 2023), which supports its validity and reliability in Spanish-speaking contexts.

### Procedure

The study protocol was reviewed and approved by the Institutional Research Ethics Committee of the Catholic University of Santa María (protocol ID: 098/2023), an ethics committee accredited and authorized to oversee research involving human participants at the national level. This approval covered data collection at all participating institutions, including public and private universities. All procedures complied with national ethical regulations and the principles of the Declaration of Helsinki. Informed consent was obtained from all participants prior to data collection. Prior to the survey, participants were informed about the objective of the study, and informed consent was obtained. Confidentiality, anonymity, and voluntary participation without incentives were ensured. The survey was self-administered and carried out virtually through the Microsoft Teams platform. After data collection, a database was created in Microsoft Excel 360, followed by coding and descriptive analysis in SPSS. Advanced statistical analyses were conducted using RStudio.

### Data analysis

Regarding analytical procedures, descriptive analyses were first conducted, reporting mean, standard deviation, skewness, and kurtosis of the items (Muñiz Fernández, 2018; Ferrando et al., 2022). Subsequently, multivariate normality was assessed. Given its violation, the Confirmatory Factor Analysis (CFA) was estimated using the Robust Maximum Likelihood method (MLR). The following model fit indices were considered: Comparative Fit Index (CFI), Tucker-Lewis Index (TLI), Root Mean Square Error of Approximation (RMSEA), and Standardized Root Mean Square Residual (SRMR). In the third stage, factorial invariance by sex was examined. Finally, internal reliability was assessed using *α* (Cronbach’s alpha) and *ω* (McDonald’s omega) coefficients, along with Average Variance Extracted (AVE) and validity related to other variables. Descriptive analyses were performed using SPSS version 22. Factorial and inferential analyses were conducted in RStudio using the Lavaan package (version 0.6–19) and the SemTools package (version 0.5–7).

Although the items were measured using a Likert-type response format, the use of the robust maximum likelihood estimator (MLR) was considered appropriate for several reasons. First, the items included five response categories, a condition under which ordinal variables can reasonably be treated as continuous without substantial bias in parameter estimation (Lloret-Segura et al., 2014; Rhemtulla et al., 2012).

Second, preliminary analyses showed acceptable univariate and multivariate distributional properties, and MLR was selected to account for residual non-normality by providing robust standard errors and corrected fit indices.

To further evaluate the robustness of the results, a sensitivity analysis was conducted using the WLSMV estimator. The factor loadings and overall model fit indices were substantively equivalent across estimation methods, supporting the use of MLR in the present study.

### Mathematical structure of the structural equation model

**Figure 1:**
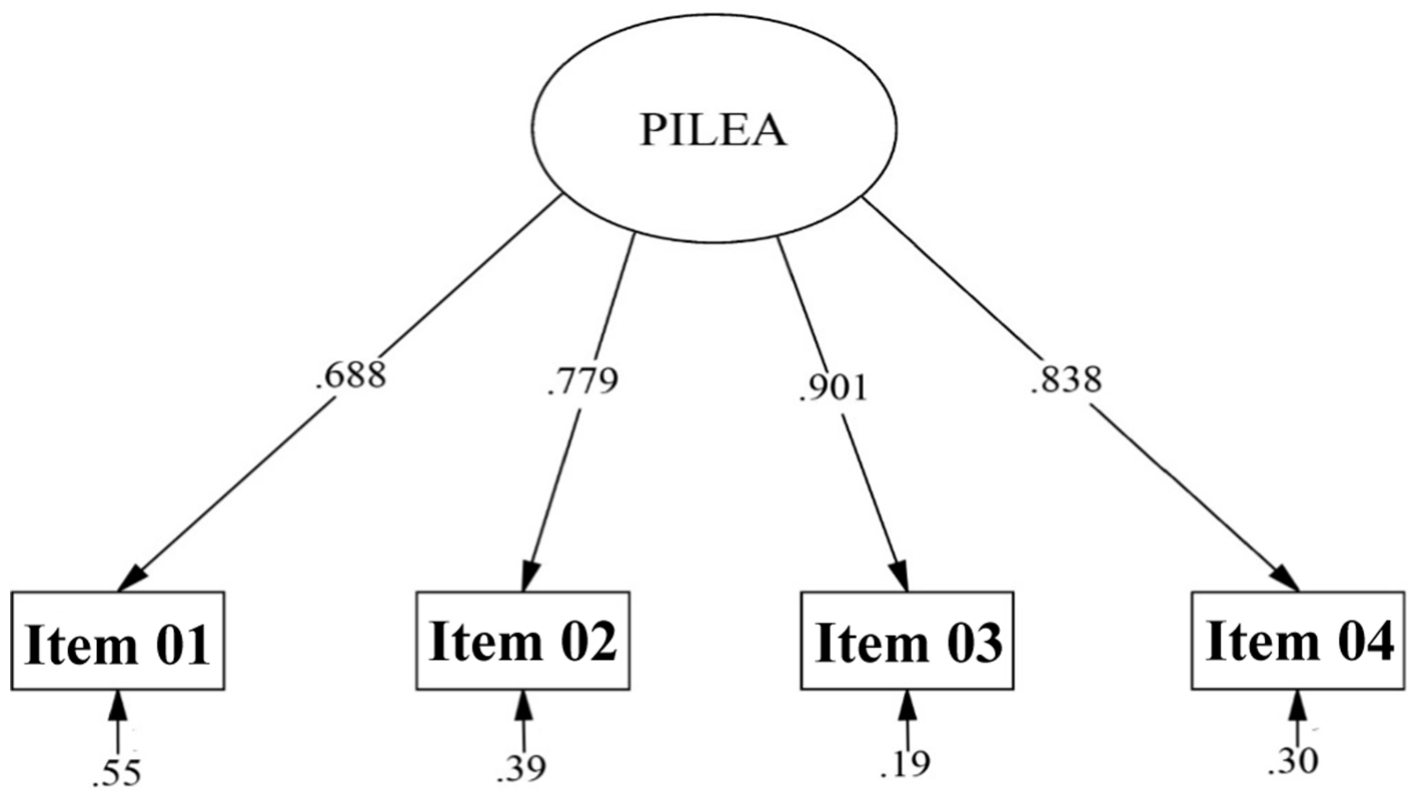
Standardized factor loadings of the CFA for the PILEA instrument. PILEA: Purpose in Life.

In order to formalize the evaluated model mathematically, Figure 1 is expressed using the standard notation for structural equation models. The model corresponds to a unidimensional measurement structure, in which a latent construct (η) explains the observed responses to the items on the Purpose in Life (PILEA-4) scale. The measurement equation is defined as: x = Λx η + δ


$$\begin{array}{ccccc} \left[x1\right] & & \left[\lambda 1\right] & & \left[\delta 1\right]\\ \left[x2\right] & = & [\lambda 2]\eta & + & \left[\delta 2\right]\\ \left[x3\right] & & \left[\lambda 3\right] & & \left[\delta 3\right]\\ \left[x4\right] & & \left[\lambda 4\right] & & \left[\delta 4\right] \end{array}$$

Where x represents the vector of observed variables (items), Λx is the vector of factor loadings, η corresponds to the latent variable Purpose of Life, and δ represents the vector of measurement errors associated with each item.

Where each observed item (x₁–x₄) is explained by the latent construct through its respective factor loading (λ₁–λ₄) and a specific error term (δ₁–δ₄). This approach allows for explicit modelling of the common variance attributable to the construct and the residual variance associated with each indicator. This matrix formulation is equivalent to the graphical representation presented in Figure 1 and forms the basis of the confirmatory factor analysis performed.

## Results

### Descriptive analysis

Regarding the descriptive analysis of the scores derived from the administration of the Purpose in Life (PILEA-4) scale, it can be observed that the lowest average score corresponds to item 3 (*M* = 3.83), and the highest average score corresponds to item 2 (*M* = 3.98), with dispersion values ranging from 0.78 to 0.87. In addition, the skewness and kurtosis values for all items were within the range of ±2, which indicates the presence of univariate normality (Tabachnick and Fidell, 2013; Field, 2024). According to these results, no ceiling or floor effects were detected, which is adequate for conducting a CFA (Table 1).

**Table 1:** Descriptive statistics of the instrument items.

Items	Min	Max	M	(SD)	g_1_	g_2_
1. My life has meaning	1	5	3.91	(0.87)	−0.76	0.85
2. My plans for the future are consistent with my true interests and values	1	5	3.98	(0.78)	−0.66	0.77
3. I know the direction my life will take	1	5	3.83	(0.86)	−0.71	0.72
4. I have clear goals that guide my life	1	5	3.90	(0.84)	−0.66	0.55

Min: minimum value; Max: maximum value; M: mean; SD: standard deviation; g_1_: skewness; g_2_: kurtosis.

### Confirmatory factor analysis

Before performing the Confirmatory Factor Analysis (CFA), multivariate normality was evaluated using Mardia’s test. The results indicated a violation of this assumption, based on the skewness (2.78, *p* < 0.001) and kurtosis (44.40, *p* < 0.001) coefficients. It should be noted that the items were treated as continuous data, as they have five response options (Lloret-Segura et al., 2014; Ferrando et al., 2022). Given this violation, and following methodological recommendations (Brown, 2015), MLR was used. The CFA results showed an adequate global fit for the unidimensional model. Although the χ^2^ statistic was significant, χ^2^(2) = 14.61, *p* < 0.05, this result should be interpreted with caution. Both the χ^2^ statistic and the χ^2^/gl ratio are known for their high sensitivity to sample size and models with a small number of degrees of freedom, which limits their usefulness as decisive fit criteria. Consequently, and following methodological recommendations in the field of structural equation modelling, the evaluation of model fit was based primarily on alternative absolute and incremental fit indices, such as CFI, TLI, SRMR, and RMSEA (Brown, 2015; Kline, 2023).

The Comparative Fit Index (CFI = 0.985) and the Tucker-Lewis Index (TLI = 0.956) exceeded the minimum cutoff criteria of 0.90 and 0.95 respectively, which is indicative of excellent model fit according to Brown (2015). Similarly, the Standardized Root Mean Square Residual (SRMR = 0.023) was below the 0.05 threshold, indicating a minimal discrepancy between observed and estimated covariances. The Root Mean Square Error of Approximation (RMSEA = 0.079; 95% CI [0.054, 0.108]) was within the range considered reasonable or acceptable (<0.08), according to established criteria (Hu and Bentler, 1999; Brown, 2015). Given the low number of degrees of freedom (df = 2), the RMSEA is interpreted with caution and in conjunction with CFI/SRMR. The results are summarized in Table 2.

**Table 2:** MLR fit indices of the CFA using the MLR estimator.

Model	*X* ^2^	df	CFI	TLI	SRMR	RMSEA	95% CI RMSEA [LL – UL]
	14.61	2	0.985	0.956	0.023	0.079	[0.054, 0.108]

X^2^: chi-square; df: degrees of freedom; CI: confidence interval; LL: lower limit, UL: upper limit.

**Figure 2:**
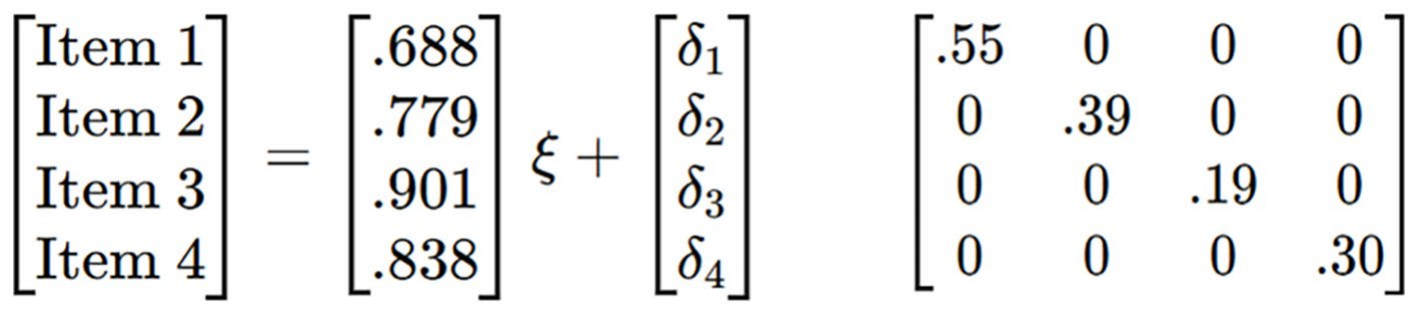
Matrix representation of the one-dimensional structural equation model of the Purpose in Life (PILEA-4) scale.

Regarding standardized factor loadings of the items, values ranged from 0.688 to 0.901 and were all statistically significant (*p* < 0.001). These loadings are considered optimal since they exceed the minimum recommended cutoff of 0.30 (Fernández Aráuz, 2015). Residuals (measurement errors) ranged from 0.19 to 0.55, which are considered low. The factor loadings and error variances for each item are shown in Figures 1, 2.

### Reliability of the instrument

To assess the reliability of the instrument, internal consistency methods were used. Cronbach’s alpha and McDonald’s omega were calculated. The alpha coefficient obtained a value of 0.873. However, based on the standardized factor loadings (see Figure 1), tau-equivalence assumptions were not met, therefore McDonald’s omega was also computed (Trizano-Hermosilla and Alvarado, 2016). The obtained value was 0.873, which is considered good (values above 0.70), indicating that the items demonstrate good internal consistency (Tavakol and Dennick, 2011). Additionally, composite reliability (CR) was calculated, resulting in 0.876, supporting the homogeneity of measurements. Regarding the Average Variance Extracted (AVE), a value of 0.642 was obtained, which indicates adequate convergent validity. When comparing the construct by sex, using the student’s *t*-test and complying with equality of variance (*p* > 0.05), male students scored higher [*t*_(df: 1004)_ = 3.412, *p* < 0.01, *d* = 0.216], although the effect size was small. These results suggest that the PILEA measures the construct Purpose in Life reliably, and that there are sex differences with a low magnitude of effect. Values are presented in Table 3.

**Table 3:** Instrument reliability.

Variable	α	ω	CR	AVE	Women	Men
					M (SD)	M (SD)
1. Purpose in Life	0.873	0.873	0.876	0.642	3.84 (0.72)	3.99 (0.70)

α: Cronbach’s alpha; ω: McDonald’s omega; CR; composite reliability, AVE: average variance extracted; M: mean; SD: standard deviation.

### Convergent validity of the instrument

To verify the convergent validity of the instrument, Pearson’s product–moment correlation coefficient was used. The Purpose in Life factor was correlated with the Positive Youth Development dimensions (the 5Cs). Table 4 shows that Purpose in Life presented positive and statistically significant correlations with each of the 5Cs. Correlations ranged from 0.188 to 0.571, representing small, moderate, and high effect sizes (Cohen, 1992; Rosenthal and Rosnow, 2008; Hernández et al., 2016).

**Table 4:** Correlation of the PILEA with the 5Cs.

Variable	*M*	SD	1	2	3	4	5	6
1. Competence	3.26	0.63	_					
2. Confidence	3.60	0.69	0.644^**^	_				
3. Character	3.77	0.45	0.342^**^	0.433^**^	_			
4. Caring	3.69	0.56	0.159^**^	0.106^**^	0.435^**^	_		
5. Connection	3.49	0.52	0.514^**^	0.537^**^	0.424^**^	0.274^**^	_	
6. Purpose in Life	3.90	0.71	0.497^**^	0.571^**^	0.380^**^	0.188^**^	0.463^**^	_

M: mean; SD: standard deviation. **p < 0.01.

Finally, a multigroup (invariance) analysis was conducted, taking into account participants’ sex. For this analysis, the minimum cutoff values suggested for differences in CFI and RMSEA were used (Cheung and Rensvold, 2002; Brown, 2015). Table 5 shows the calculated fit indices and their respective differences. It can be observed that invariance is supported up to the strict level, since all levels of measurement invariance (configural, metric, scalar, and strict) show changes of ≤0.01 in CFI and ≤0.015 in RMSEA. These results indicate that the PILEA instrument has a similar factorial structure, factor loadings, intercepts, and error variances in both the male and female groups. Given that scalar and strict invariance were established, latent mean differences were subsequently examined. The female group was used as the reference group (fixing its latent mean to 0). Results showed that men had higher latent means than women (LM_diff_ = −0.242, SE = 0.064, *p* < 0.01), although with a small effect size (*d*_Glass_ = 0.235).

**Table 5:** Measurement invariance analysis by sex.

Models	Sex (Women and Men)					
	*X* ^2^	df	CFI	RMSEA	∆CFI	∆RMSEA
1. Configural	23.80**	4	0.979	0.099		
2. Metric	31.08**	7	0.974	0.083	−0.005	−0.016
3. Scalar	37.63**	10	0.970	0.074	−0.004	−0.009
4. Strict	45.99**	14	0.966	0.067	−0.004	−0.007

ΔCFI and ΔRMSEA are reported relative to the previous model. Criteria: ΔCFI ≤ 0.010; ΔRMSEA ≤ 0.015. **p < 0.01.

The multigroup analysis confirmed invariance by sex at the configural, metric, scalar, and strict levels. The changes between models were small (ΔCFI and ΔRMSEA met the recommended criteria), supporting the equivalence of the measure between women and men. Once scalar invariance was confirmed, latent means were compared, and small effect size differences were observed, so their practical relevance should be considered modest.

## Discussion and conclusion

Our results confirm that the PILEA-4 shows a unidimensional structure with good fit (CFI ≈ 0.985; TLI ≈ 0.956; SRMR ≈ 0.023) and high internal consistency (*ω* ≈ 0.87), as well as convergent validity (AVE ≈ 0.64; correlations with the 5Cs *r* ≈ 0.19–0.57). Measurement invariance by sex was achieved at the strict level, which allows valid comparisons between women and men. Overall, these findings support the use of the PILEA-4 as a brief measure suitable for Peruvian university contexts, where purpose in life is linked to Positive Youth Development and the 5Cs model, which includes psychosocial competencies relevant to wellbeing in emerging adulthood (Hill et al., 2016).

Compared to the original validation in emerging adults (Hill et al., 2016), our standardized loadings (≈ 0.69–0.90) and reliability (ω ≈ 0.87) are equal to or higher than those initially reported (*α* ≈ 0.81), and the convergence (AVE ≈ 0.64) supports the functional unidimensionality of the construct in this population. The fit indices (CFI ≈ 0.99; TLI ≈ 0.96; SRMR ≈ 0.02; RMSEA ≈ 0.08, with df = 2) are consistent with a brief structure and, despite the sensitivity of χ^2^/df, are supported by robust indices. Taken together, these results extend the evidence to a Latin American context, maintaining the parsimony of the model.

These results are consistent with the brief original validation of Purpose in Life in emerging adults (Hill et al., 2016), in which purpose was conceptualized as a central and self-organized life aim associated with identity indicators and adaptive functioning. The present study contributes to the evidence on the reliability and validity of purpose scales in Spanish-speaking populations (Rubio-Belmonte et al., 2022), specifically in the Latin American context, suggesting cross-cultural invariance (Caycho-Rodríguez et al., 2022). Likewise, the positive association with PYD indicators is consistent with studies that consider Purpose in Life as a developmental asset in youth, connecting it with eudaimonic wellbeing (Lund et al., 2024; Rojas Zegarra et al., 2025).

From an applied perspective, assessing purpose using the PILEA-4 makes it possible to identify students with low levels of Purpose in Life and to guide actions for the implementation of psychoeducational programs designed to promote wellbeing of university students. The literature suggests that Purpose in Life is associated with better mental health and therefore lower levels of anxiety and depression (Boreham and Schutte, 2023), and that it can be fostered through relational supports and high-impact educational experiences, such as service-learning, in university environments (Lund et al., 2024). In adolescents and youth, Purpose in Life acts as a developmental asset and a protective factor against emotional distress (Barcaccia et al., 2023), reinforcing its preventive value in this population.

Methodologically, it is important to interpret RMSEA with caution in models with few degrees of freedom, prioritizing its interpretation in conjunction with CFI and SRMR (Kenny et al., 2014).

This study has some limitations. One of them relates to the sampling procedure, which was based on convenience sampling. Another limitation is the absence of temporal stability evidence (test–retest). For future studies, it is recommended to estimate and report intraclass correlation coefficients with their 95% CI (Koo and Li, 2016; Terwee et al., 2018). In addition, no item-level DIF analyses were performed within a measurement invariance framework (Millsap, 2012), and no formal assessment of discriminant validity was conducted, for example using the HTMT criterion (Henseler et al., 2015). It is also necessary to consider the possible common method bias inherent to self-report measures (Podsakoff et al., 2003). Furthermore, non-probabilistic convenience sampling in a single city limits external validity; multicenter studies with probabilistic sampling will allow for more accurate estimation of the generalization of measurement parameters. In addition to anonymity and consent, we recommend procedural remedies (instructions that reduce social evaluation, random order of scales) and analytical remedies (e.g., common method bias or marker item) when the design allows.

Future research should employ multicenter probabilistic sampling and examine invariance associated with type of university and age groups, as well as intervention trials aimed at strengthening purpose (e.g., guided writing about valued goals, purpose-based mentoring, and service-learning). Reports that integrate test–retest, SEM/SDC, and discriminant validity according to COSMIN standards would improve the diagnostic and predictive utility of the PILEA-4. In summary, the scale provides solid psychometric evidence for its use in university contexts, with concrete implications for the promotion of wellbeing.

The PILEA-4 showed a unidimensional structure with good fit, high internal consistency, and convergent validity with Positive Youth Development indicators (5Cs). It also showed measurement invariance by sex up to the strict level, which enables valid comparisons between women and men. Taken together, these findings support the use of the PILEA-4 as a brief measure of Purpose in Life in Peruvian university populations.

In practical terms, PILEA-4 can be integrated into (a) tutorials to identify students who lack purpose in life and agree on personalized objectives; (b) purpose-driven mentoring oriented toward values and academic meaning; (c) service learning to align social contribution and vocational trajectory; and (d) short curricular modules (e.g., writing valuable goals and guided reflection). These applications facilitate semester-long (pre–post) follow-up and referral to psychoeducational supports when appropriate. It could also help prevent university dropouts and mental health problems that arise in this vulnerable population.

In applied terms, having a concise and valid instrument allows the integration of Purpose in Life into psychoeducational programs, tutoring, mentoring, and service-learning initiatives, with the potential to reduce risks associated with psychological distress and to strengthen developmental assets.

## Data Availability

The raw data supporting the conclusions of this article will be made available by the authors, without undue reservation.

## Appendix: Formulas for calculating goodness-of-fit indices

*Robust chi-square of* Satorra and Bentler (2001):

$$X_{\mathrm{rob}}^{2}=\frac{T}{\hat{c}}$$

Where: T is the standard chi-square, ĉ is the scale factor.

*Model Null:*

$$X_{\mathrm{rob},\text{null}}^{2}=\frac{T_{\text{null}}}{{\hat{c}}_{\text{null}}}$$

*Robust CFI calculation for MLR*

$$\mathrm{CF}I_{\mathrm{rob}}=1-\frac{X_{\mathrm{rob},\text{model}}^{2}-gl_{\text{model}}}{X_{\mathrm{rob},\text{zero}}^{2}-gl_{\text{zero}}}$$

*Robust TLI calculation for MLR*

$$\mathrm{TL}I_{\mathrm{rob}}=\frac{\left(\frac{X_{\mathrm{rob},\text{null}}^{2}}{gl_{\text{nulo}}})-(\frac{X_{\mathrm{rob},\text{model}}^{2}}{gl_{\text{modelo}}}\right)}{(\frac{X_{\mathrm{rob},\text{null}}^{2}}{gl_{\text{zero}}})-1}$$

Robust RMSEA calculation for MLR

$$\text{RMSE}A_{\mathrm{rob}}=\sqrt{\frac{\max(X_{\mathrm{rob},\text{model}}^{2}-gl_{\text{model}}.0}{gl_{\text{model}}(N-1)}}$$

SRMR calculation

$$\text{SRMR}=\sqrt{(\frac{1}{\frac{p(p+1)}{2}}).\sum_{i<j}{\left(r_{\mathrm{ij}}-{\overset{⌣}{r}}_{\mathrm{ij}}\right)}^{2}}$$

Where: p is the number of observed variables, r_ij are observed correlations, and ř_ij are the correlations reproduced by the model.

Calculation of AVE

$$\mathrm{AVE}=\frac{\sum_{i=1}^{k}\lambda_{i}^{2}}{\sum_{i=1}^{k}\lambda_{i}^{2}+\sum_{i=1}^{k}\theta_{i}}$$

Where *λ*₁ are the factorial loadings and *ϴ*₁ are the item error variances.

## References

[ref1] Abdul Kadir N. B. Mohd R. H. (2021). The 5Cs of positive youth development, purpose in life, Hope, and well-being among emerging adults in Malaysia. Front. Psychol. 12:641876. doi: 10.3389/fpsyg.2021.641876, 34335359 PMC8319496

[ref2] Bakhiyi C. L. Calati R. Guillaume S. Courtet P. (2016). Do reasons for living protect against suicidal thoughts and behaviors? A systematic review of the literature. J. Psychiatr. Res. 77, 92–108. doi: 10.1016/j.jpsychires.2016.02.019, 27014850

[ref3] Barcaccia B. Couyoumdjian A. Di Consiglio M. Papa C. Cancellieri U. G. Cervin M. (2023). Purpose in life as an asset for well-being and a protective factor against depression in adolescents. Front. Psychol. 14:1250279. doi: 10.3389/fpsyg.2023.1250279, 37829070 PMC10566624

[ref9001] Batanova M. Weissbourd R. McIntyre J. (n.d.). Loneliness in America: Just the Tip of the Iceberg?, 37572371

[ref4] Boreham I. D. Schutte N. S. (2023). The relationship between purpose in life and depression and anxiety: a meta-analysis. J. Clin. Psychol. 79, 2736–2767. doi: 10.1002/jclp.23576, 37572371

[ref5] Brown T. A. (2015). Confirmatory factor analysis for applied research, second edition. New York, NY: Guilford Publications.

[ref6] Campbell A. R. Hill P. L. Nicholson V. Lambert S. Cote H. C. F. Edmonds G. W. . (2024). Exploring sense of purpose and conscientiousness as correlates to health and well-being with indigenous and low socioeconomic communities on coast Salish territories, Vancouver, Canada. Can. J. Behav. Sci. Rev. Can. Sci. Comport. 56, 240–252. doi: 10.1037/cbs0000363, 39131185 PMC11313659

[ref7] Caycho-Rodríguez T. Vilca L. W. Cervigni M. Gallegos M. Martino P. Calandra M. . (2022). Cross-national measurement invariance of the purpose in life test in seven Latin American countries. Front. Psychol. 13:974133. doi: 10.3389/fpsyg.2022.974133, 36186323 PMC9524452

[ref8] Cheung G. W. Rensvold R. B. (2002). Evaluating goodness-of-fit indexes for testing measurement invariance. Struct. Equ. Model. 9, 233–255. doi: 10.1207/S15328007SEM0902_5

[ref9] Cohen J. (1992). A power primer. Psychol. Bull. 112, 155–159. doi: 10.1037/0033-2909.112.1.155 19565683

[ref10] Damon W. Menon J. Bronk K. C. (2003). The development of purpose during adolescence. Appl. Dev. Sci. 7, 119–128. doi: 10.1207/s1532480xads0703

[ref11] Ehsani J. P. Duren M. L. Grant B. J. B. Musci R. J. Eshragi A. C. Koppel S. (2024). A positive psychology framework for understanding teenage driving behaviors: examining the role of life purpose and mindfulness. Traffic Inj. Prev. 25, S1–S5. doi: 10.1080/15389588.2024.2372782, 39485700

[ref12] Fernández Aráuz A. (2015). Aplicación del análisis factorial confirmatorio a un modelo de medición del rendimiento académico en lectura. Rev. Cienc. Econ. 33, 39–65. doi: 10.15517/rce.v33i2.22216

[ref13] Ferrando P. Lorenzo-Seva U. Hernández-Dorado A. Muñiz J. (2022). Decalogue for the factor analysis of test items. Psicothema 34, 7–17. doi: 10.7334/psicothema2021.456, 35048890

[ref14] Field A. (2024). Discovering statistics using IBM SPSS statistics. London: Sage Publications Limited.

[ref15] Frankl V. (2011). La voluntad de sentido: conferencias escogidas sobre logoterapia. Herder editorial. Available online at: https://books.google.com/books?hl=es&lr=&id=BAKIDwAAQBAJ&oi=fnd&pg=PT36&dq=Frankl,+V.+E.+(1994).+La+voluntad+de+sentido.+Conferencias+escogidas+sobre+logoterapia.+Herder&ots=RoPt0nY0St&sig=HOUc5acZk510LV_NL5Q9gxjB2FA (Accessed October 2, 2025)

[ref16] Geldhof G. J. Bowers E. P. Boyd M. J. Mueller M. K. Napolitano C. M. Schmid K. L. . (2014). Creation of short and very short measures of the five Cs of positive youth development. J. Res. Adolesc. 24, 163–176. doi: 10.1111/jora.12039

[ref17] Gómez-Tabares A. S. Restrepo J. E. Hincapié Aguirre N. González- Pérez A. (2024). The mediating role of purpose in life in the relationship between hopelessness, depression, and suicide risk. Mediterr. J. Clin. Psychol. (2024) 12, 1–22. doi: 10.13129/2282-1619/MJCP-4047

[ref18] Henseler J. Ringle C. M. Sarstedt M. (2015). A new criterion for assessing discriminant validity in variance-based structural equation modeling. J. of the Acad. Mark. Sci. 43, 115–135. doi: 10.1007/s11747-014-0403-8

[ref19] Hernández A. Ponsoda V. Muñiz J. Prieto G. Elosua P. (2016). Revisión del Modelo Para evaluar la calidad de los tests utilizados en España. [Assessing the quality of tests in Spain: revision of the Spanish test review model.]. Pap. Psicol. 37, 192–197.

[ref20] Hill P. L. Edmonds G. W. Hampson S. E. (2019). A purposeful lifestyle is a healthful lifestyle: linking sense of purpose to self-rated health through multiple health behaviors. J. Health Psychol. 24, 1392–1400. doi: 10.1177/1359105317708251, 28810459 PMC5665713

[ref21] Hill P. L. Edmonds G. W. Peterson M. Luyckx K. Andrews J. A. (2016). Purpose in life in emerging adulthood: development and validation of a new brief measure. J. Posit. Psychol. 11, 237–245. doi: 10.1080/17439760.2015.1048817, 26958072 PMC4779362

[ref22] Hu L. Bentler P. M. (1999). Cutoff criteria for fit indexes in covariance structure analysis: conventional criteria versus new alternatives. Struct. Equ. Model. 6, 1–55. doi: 10.1080/10705519909540118

[ref23] Kenny D. A. Kaniskan B. McCoach D. B. (2014). The performance of RMSEA in models with small degrees of freedom. Sociol. Methods Res. 44, 486–507. doi: 10.1177/0049124114543236

[ref24] Kleiman E. M. Beaver J. K. (2013). A meaningful life is worth living: meaning in life as a suicide resiliency factor. Psychiatry Res. 210, 934–939. doi: 10.1016/j.psychres.2013.08.002, 23978733

[ref25] Kline R. B. (2023). Principles and practice of structural equation modeling. New York, NY: Guilford Publications.

[ref26] Koo T. K. Li M. Y. (2016). A guideline of selecting and reporting Intraclass correlation coefficients for reliability research. J. Chiropr. Med. 15, 155–163. doi: 10.1016/j.jcm.2016.02.012, 27330520 PMC4913118

[ref27] Lloret-Segura S. Ferreres-Traver A. Hernández-Baeza A. Tomás-Marco I. (2014). Exploratory item factor analysis: a practical guide revised and updated. An. Psicol. 30, 1151–1169. doi: 10.6018/analesps.30.3.199361

[ref28] Lund T. J. Fongemy G. Lincoln B. Snow H. Hakovirta A. l. S. Liang B. (2024). Predictors of purpose among young adults in college: an exploratory analysis of the importance of relational supports and experiential learning. Youth 4, 1494–1504. doi: 10.3390/youth4040095

[ref29] Manrique-Millones D. Gómez-Baya D. Wiium N. (2023). The importance of the 5Cs of positive youth development to depressive symptoms: a cross-sectional study with university students from Peru and Spain. Behav. Sci. 13:280. doi: 10.3390/bs13030280, 36975305 PMC10045354

[ref30] Marín-Gutiérrez M. Caqueo-Urízar A. Castillo-Francino J. Escobar-Soler C. (2024). The 5Cs of positive youth development: their impact on symptoms of depression, anxiety, stress, and emotional distress in Chilean adolescents. BMC Psychol. 12:372. doi: 10.1186/s40359-024-01863-x, 38951933 PMC11218329

[ref31] McKnight P. E. Kashdan T. B. (2009). Purpose in life as a system that creates and sustains health and well-being: an integrative, testable theory. Rev. Gen. Psychol. 13, 242–251. doi: 10.1037/a0017152

[ref32] Millsap R. E. (2012). Statistical approaches to measurement invariance. New York: Routledge.

[ref33] Muñiz Fernández J. (2018). *Introducción a la psicometría: teoría clásica y TRI*. Madrid: Pirámide. Available at: https://digibuo.uniovi.es/dspace/handle/10651/54694 (Accessed November 27, 2025).

[ref34] Pérez-Díaz P. A. Ardiles-Irarrázabal R.-A. Schoeps K. Valero-Moreno S. Wiium N. (2024). Positive identity and connection on purpose in life in Chilean youth. Rev. Latinoam. Psicol. 56, 242–250. doi: 10.14349/rlp.2024.v56.24

[ref35] Podsakoff P. M. MacKenzie S. B. Lee J.-Y. Podsakoff N. P. (2003). Common method biases in behavioral research: a critical review of the literature and recommended remedies. J. Appl. Psychol. 88, 879–903. doi: 10.1037/0021-9010.88.5.879, 14516251

[ref9003] Rhemtulla M. Brosseau-Liard P. É. Savalei V. (2012). When can categorical variables be treated as continuous? A comparison of robust continuous and categorical SEM estimation methods under suboptimal conditions. Psychol. Methods 17, 354–373. doi: 10.1037/a0029315, 22799625

[ref36] Rojas Zegarra M. E. Pérez-Díaz P. A. Yana Calla V. R. Vilca-Pareja V. Ardiles-Irarrázabal R.-A. Muñoz-del-Carpio-Toia A. (2025). Positive youth predictors of purpose in life in Peruvian university students. BMC Psychol 13:782. doi: 10.1186/s40359-025-03116-x, 40652281 PMC12255062

[ref37] Rosenthal R. Rosnow R. L. (2008). *Essentials of Behavioral Research: Methods and Data Analysis (3rd edition)*. Available online at: http://hdl.handle.net/20.500.12613/79 (Accessed October 5, 2025).

[ref38] Rubio-Belmonte C. Mayordomo-Rodríguez T. García-Alandete J. (2022). Psychometric properties of the purpose in life-short form in the Spanish population. J. Clin. Psychol. 79, 1099–1112. doi: 10.1002/jclp.23461, 36417619

[ref39] Ryff C. D. (2014). Self-realisation and meaning making in the face of adversity: a eudaimonic approach to human resilience. J. Psychol. Afr. 24, 1–12. doi: 10.1080/14330237.2014.904098, 25435804 PMC4243302

[ref40] Ryff C. D. Keyes C. L. M. (1995). The structure of psychological well-being revisited. J. Pers. Soc. Psychol. 69, 719–727. doi: 10.1037/0022-3514.69.4.719, 7473027

[ref9002] Satorra A. Bentler P. M. (2001). A Scaled Difference Chi-Square Test Statistic for Moment Structure Analysis. Psychometrika. 66, 507–514. doi: 10.1007/BF022961, 20640194 PMC2905175

[ref41] Sharma G. Yukhymenko-Lescroart M. Sanchez T. (2023). Examining the role of life purpose in high school students’ self-esteem through structural equation modelling. Heliyon 9:e19614. doi: 10.1016/j.heliyon.2023.e19614, 37809688 PMC10558860

[ref42] Soper D. S. n.d.. A-priori sample size calculator for structural equation models. Available online at: https://www.danielsoper.com/statcalc/calculator.aspx?id=89 (Accessed January 27, 2026).

[ref43] Tabachnick B. Fidell (2013). Using multivariate statistics (6th ed.). Pearson Education - Buscar con Google. Available online at: http://ndl.ethernet.edu.et/bitstream/123456789/27657/1/Barbara%20G.%20Tabachnick_2013.pdf (Accessed October 2, 2025).

[ref44] Tavakol M. Dennick R. (2011). Making sense of Cronbach’s alpha. Int. J. Med. Educ. 2, 53–55. doi: 10.5116/ijme.4dfb.8dfd, 28029643 PMC4205511

[ref45] Terwee C. B. Prinsen C. A. C. Chiarotto A. Westerman M. J. Patrick D. L. Alonso J. . (2018). COSMIN methodology for evaluating the content validity of patient-reported outcome measures: a Delphi study. Qual. Life Res. 27, 1159–1170. doi: 10.1007/s11136-018-1829-0, 29550964 PMC5891557

[ref46] Trizano-Hermosilla I. Alvarado J. M. (2016). Best alternatives to Cronbach’s alpha reliability in realistic conditions: congeneric and asymmetrical measurements. Front. Psychol. 7:769. doi: 10.3389/fpsyg.2016.00769 27303333 PMC4880791

[ref47] Zhu M. Zhang W. Jiang F. (2024). How to influence and cultivate young adults’ life purpose in the process of education: a systematic review of empirical studies. BMC Psychol. 12:554. doi: 10.1186/s40359-024-02003-1, 39407292 PMC11481473

